# B Cell Abnormalities in Systemic Lupus Erythematosus and Lupus Nephritis—Role in Pathogenesis and Effect of Immunosuppressive Treatments

**DOI:** 10.3390/ijms20246231

**Published:** 2019-12-10

**Authors:** Desmond Y. H. Yap, Tak Mao Chan

**Affiliations:** Division of Nephrology, Department of Medicine, Queen Mary Hospital, The University of Hong Kong, Hong Kong; dtmchan@hku.hk

**Keywords:** B cell abnormalities, systemic lupus erythematosus, lupus nephritis, pathogenesis, treatment

## Abstract

Abnormalities in B cells play pivotal roles in the pathogenesis of systemic lupus erythematosus (SLE) and lupus nephritis (LN). Breach in central and peripheral tolerance mechanisms generates autoreactive B cells which contribute to the pathogenesis of SLE and LN. Dysregulation of B cell transcription factors, cytokines and B cell–T cell interaction can result in aberrant B cell maturation and autoantibody production. These immunological abnormalities also lead to perturbations in circulating and infiltrating B cells in SLE and LN patients. Conventional and novel immunosuppressive medications confer differential effects on B cells which have important clinical implications. While cyclophosphamide and mycophenolate mofetil (MMF) showed comparable clinical efficacy in active LN, MMF induction was associated with earlier reduction in circulating plasmablasts and plasma cells. Accumulating evidence suggests that MMF maintenance is associated with lower risk of disease relapse than azathioprine, which may be explained by its more potent and selective suppression of B cell proliferation. Novel therapeutic approaches targeting the B cell repertoire include B cell depletion with monoclonal antibodies binding to cell surface markers, inhibition of B cell cytokines, and modulation of costimulatory signals in B cell–T cell interaction. These biologics, despite showing improvements in serological parameters and proteinuria, did not achieve primary endpoints when used as add-on therapy to standard treatments in active LN patients. Other emerging treatments such as calcineurin inhibitors, mammalian target of rapamycin inhibitors and proteasome inhibitors also show distinct inhibitory effects on the B cell repertoire. Advancement in the knowledge on B cell biology has fueled the development of new therapeutic strategies in SLE and LN. Modification in background treatments, study endpoints and selective recruitment of subjects showing aberrant B cells or its signaling pathways when designing future clinical trials may better elucidate the roles of these novel therapies for SLE and LN patients.

## 1. Introduction

Systemic lupus erythematosus (SLE) is an autoimmune disease with abnormal interplay between innate and adaptive immunity, breach of immune tolerance, production of autoantibodies, and immunological insult to multiple organ systems. Kidney involvement is common among patients with SLE, and the presence of lupus nephritis (LN) significantly increased the risk of renal failure and patient mortality [1,2]. The management of LN is challenging because of substantial patient variability in disease course and response to treatment [3,4]. Such patient heterogeneity may be related to the complexity of LN pathogenesis. Genetic predispositions, abnormalities in lymphocytes, aberrant complement activation, autoantibody production, and perturbed cytokine milieu all contribute to the pathogenesis of SLE and LN [5,6,7]. In this context, abnormalities in B cells is a key player in SLE and LN pathogenesis as autoantibodies are important for diagnosis and the changes in autoantibodies level may also show correlations with clinical disease activity [5,6,7,8,9,10]. B cells also show important immunological functions pertinent to SLE and LN pathogenesis such as presentation of autoantigens and secretion of proinflammatory cytokines. Therefore, the study of B lymphocytes can potentially unravel important pathogenic mechanisms of SLE/LN and thus help develop more specific therapies to improve treatment efficacy and tolerability. The following discussion will highlight the B cell abnormalities which are relevant to SLE and LN pathogenesis and also the effect of immunosuppressive medications on the B cell repertoire.

## 2. Normal Development and Homeostasis of B Lymphocytes

The development of B cells begins in the bone marrow. Hematopoietic stem cells commit to the lymphoid lineage and become common lymphoid precursors (CLP) [11]. CLP differentiate into pre-B cells and pro-B cells, which will then undergo immunoglobulin heavy and light chain gene rearrangement to form B cell receptors (BCR) and surface Immunoglobulin M (IgM) to become immature B cells. These stages of B cell development within the bone marrow are antigen-independent. Immature B cells exit the bone marrow to complete further maturation and differentiation in peripheral secondary lymphoid organs such as the spleen or lymph nodes, whereby B cells can be activated by either T cell-dependent or -independent pathways [12]. Within the secondary lymphoid organs, immature B cells migrate towards the lymphoid follicles. The majority of naïve B cells move into the germinal center (follicular B cells) while a small number of naïve B cells remain in the marginal zone. Follicular B cells, after cognate interaction with T helper cells, will undergo somatic hypermutation (SHM) and class switch recombination (CSR) to enhance the affinity of immunoglobulins [11,12]. Follicular B cells can further differentiate into memory B cells when the antigenic stimulation is relatively weak but into long-lived plasma cells when there are strong antigenic stimuli. Memory B cells usually remain in a dormant state, but can rapidly differentiate into antibody-producing effector cells when re-challenged with previously encountered antigens. Long-lived plasma cells mostly migrate back to the bone marrow and are responsible for antibody production, although a small proportion will remain in peripheral tissues [13]. In contrast, naïve B cells which remain in the marginal zone will become short-lived plasma cells.

The homeostasis of B cells is tightly orchestrated by various transcription factors and cytokines., The early B cell factor (EBF) promotes CLP to commit to the B cell lineage and initiates immunoglobulin heavy chain rearrangement [14]. Paired box protein -5 (PAX5) is a master regulator in B cell development at different stages, and regulates gene transcription by recruiting chromatin-remodeling, histone-modifying, and basal transcription factor complexes to its target genes [12]. PAX5 shows dual effects on early B cell development, which include the suppression of B-lineage-inappropriate genes and activation B cell-specific genes [15]. PAX5 is also involved in V(D)J recombination, B cell signaling, as well as adhesion and migration of B lymphocytes [15]. BACH2 is another signature B cell transcription factor with instrumental roles in B lymphocyte development starting early from the CLP stage. broad complex-tramtrack-bric a brac and Cap’n’collar homology 1 (BACH2), with broad complex-tramtrack-bric a brac and Cap’n’collar homology 2 (BACH1) as an auxiliary, suppress ‘myeloid genes’ in pre-pro-B cells by binding to their regulatory regions to promote development of B lymphocytes [16]. BACH2 expression is also critical in determining the fate of B cells during germinal center reaction and can interact with BCL-6 and repress lymphocyte-induced maturation protein-1 (BLIMP-1) transcription to prevent premature plasma cell differentiation [17]. Murine splenic B cells in the absence of BACH2 show increased propensity to differentiate into plasma cells, and such differentiation is regulated via both BLIMP-1-dependent and -independent pathways [18]. On the other hand, B lymphocytes which show increased BACH2 and decreased BLIMP-1 in the germinal center will preferentially differentiate into memory B cells [14].

The survival, maturation and differentiation of B lymphocytes are also affected by various B cell-related cytokines such as B-cell activating factor (BAFF), interleukin-6 (IL-6) and interleukin (IL-21) [19]. Both BAFF (also known as B lymphocytes stimulator, BLys) and a proliferation-inducing ligand (APRIL) belong to the TNF ligand superfamily and are actively secreted by dendritic cells, macrophages, and neutrophils. BAFF and APRIL are key survival factors for B lymphocytes and have potent stimulatory effects on B cell proliferation and antibody production [20]. BAFF can bind to three types of receptors: BAFF receptor, transmembrane activator, and calcium modulator and cyclophilin ligand interactor (TACI) and B-cell maturation antigen (BCMA), while APRIL can only bind to TACI and BCMA [19,21,22]. Most B cell subsets express all three types of receptors but plasma cells only show BCMA on their cell surface [19,21]. These variations in receptor expression may account for the differential response of B cell subsets to biologics targeting B cell survival factors. IL-6 is a proinflammatory cytokine produced by monocytes, fibroblasts, and endothelial cells, and to a lesser extent B and T cells and resident renal cells [19]. IL-6 promotes the maturation of naïve B cells into memory or plasma cells, differentiation of follicular T helper cells (T_FH_), formation of germinal center, and production of antibodies (including autoantibodies) [19,23]. IL-21 is a cytokine produced by activated T helper cells (especially Th2, Th17, and T_FH_ cells), and exerts strong driving effects on plasma cell proliferation and differentiation as well as immunoglobulin production [19]. In this context, IL-21 works synergistically with CD40L to induce differentiation of naïve and memory B cells into antibody-producing plasma cells [24]. The role of these B cell-related cytokines in SLE and LN pathogenesis and the implications on treatment will be further discussed in the following sections.

## 3. Abnormalities in B Cell Tolerance and Regulation—Role in SLE and LN Pathogenesis

The development and survival of B cells which react against self-antigens are prevented by both central and peripheral tolerance mechanisms [14]. Failure of these tolerance mechanisms will result in generation of autoreactive B cells which contribute to the pathogenesis of SLE and LN. Abnormality in BCR signaling is one important mechanism for the breakdown of central tolerance. Deficiency of surrogate light chains (SLC) was associated with development of autoreactive B cells, possibly as a result of the selection advantage of BCR expressing self-recognizing IgH chains [14]. Impaired BCR signaling in B cells will also lead to failure to trigger apoptosis of early B cells which recognize self-antigens [14,25].

Defects in peripheral B cell tolerance are also pertinent to B cell autoimmunity. SLE patients show aberrant and increased expression of recombination-activating genes (RAG) in peripheral B cells, which can lead to mutation of BCR and thus development of autoreactive B lymphocytes [26]. Aberrant T cell-B cell interaction (e.g., shorted interaction time in the germinal center) results in inadequate anergic signals to autoreactive B cells and hence enhancing their survival [27,28]. Increased BCR-mediated signaling can also lower the activation threshold of peripheral B lymphocytes and promote cellular phenotypes characteristic of SLE [29]. Lupus B cells also showed augmented SHM and CSR [30,31,32,33,34], which contribute to increased pathogenicity of plasma cells. Other immunological anomalies relevant to B cell autoreactivity in SLE include increased plasma cell differentiation and survival, upregulated Toll-like receptor (TLR) signaling and increased expression of key B cell cytokines such as BAFF, IL-6, and IL-21 [13,35,36,37,38,39,40]. Indeed, transgenic mice overexpressing BAFF exhibited increased number of plasma cells in secondary lymphoid tissues, presence of autoantibodies, and also increased circulating immune complexes and immunoglobulin deposition in the kidneys [41]. Raised blood levels of BAFF has been observed in NZB/W F1 and MRL/*lpr* mice at the onset of disease [22], and treatment with soluble TACI-Ig mitigated the development of proteinuria and improved survival of NZB/W F1 mice [22]. Deletion of TACI receptor in transgenic mice overexpressing BAFF inhibited immune activation, diminished immunoglobulins production and conferred long-term protection from progressive glomerulonephritis for up to 12 months in these mice [42]. Elevated circulating BAFF levels have been observed in patients with SLE, which correlated with anti-dsDNA autoantibody levels and SLEDAI scores [43]. 

Interleukin-6 (IL-6) is a proinflammatory cytokine and its strong pathogenic significance in SLE and LN has been demonstrated by both animal and human studies. B lymphocytes isolated from SLE patients secrete high amount of IL-6 which can bind to the IL-6 receptor of other B cells to promote their terminal differentiation, and thus forming a positive IL-6 feedback loop [44]. Treatment with polyclonal anti-IL-6 or anti-IL-6 receptor monoclonal antibodies could inhibit IL-6 binding and suppressed total IgG and IgG anti-ssDNA antibody secretion in lupus B cells [44]. In a murine SLE model, B cell-derived IL-6 could induce T_FH_ differentiation and initiate germinal center formation [45]. Treatment of lupus prone NZB/W F1 mice with IL-6 exacerbated glomerulonephritis [46], whilst treatment with anti-IL-6 monoclonal antibodies in NZB/W F1 mice ameliorated kidney manifestations and reduced circulating anti-dsDNA autoantibodies titers [47,48]. Active LN patients showed elevated urinary levels of IL-6 compared with patients in remission [49], and renal biopsies obtained from LN patients also showed increased IL-6 expression in the glomerular and tubular regions [50]. 

IL-21 is a key driver of plasma cell differentiation and proliferation and thus has important pathogenic relevance in SLE. B lymphocytes isolated from SLE patients, when stimulated with autologous CD3^+^ T lymphocytes and IL-21, showed prominent increase in IgG production whereas treatment with Fc fusion protein against IL-21 receptor (IL-21R) would inhibit the differentiation of B lymphocytes into plasma cells [51]. BXSB-Yaa lupus-prone mice showed higher circulating IL-21 and its mRNA transcripts compared with wild-type mice [52], and deletion of IL-21R would abrogate characteristic lupus phenotypes such as autoantibodies production and glomerulonephritis in these mice [53]. Treatment of MRL/lpr mice with IL-21R.Fc fusion protein reduced anti-dsDNA autoantibody titers and lymph node enlargement, and also alleviated renal and dermatological lesions [54]. SLE patients showed raised serum IL-21 levels, and population-based case-control association analysis demonstrated that genetic polymorphisms in the IL-21 (rs907715) and IL-21R gene (rs2221903) were associated with escalated risk of SLE in European-American patients [55,56]. 

Toll-like receptors (TLR) play pivotal roles in B cell activation and also contribute to the pathogenesis of SLE and LN. In this context, TLR-7 and TLR-9 are potent inducers of Type I interferon response and show more pathogenic relevance in SLE and LN [57]. TLR-7 is expressed on different B cell subpopulations and a previous study showed that autophagy in B cells was a trigger for TLR-7-dependent autoantibody production [58,59]. BCR-driven uptake of immune complexes stimulates TLR-7 and -9 in B cells and promotes RNA- and DNA-autoantibodies production [39,60,61,62,63]. TLR-9 signaling in B lymphocytes is also essential for generation of autoantibodies against DNA in mice and enhances the differentiation of autoantibody-producing B cells and plasma cells in human [64,65]. TLR-9 mRNA expression was also increased in PBMCs isolated from SLE patients and correlated with severity of LN and anti-DNA antibody titers [66].

## 4. Perturbations in Circulating and Infiltrating B Cell Subsets—Role in SLE and LN Pathogenesis

Abnormalities in the tolerance and regulatory mechanisms of the B cell repertoire in SLE and LN can result in perturbations in the B lymphocyte subsets and their immune responsiveness. B cells which secrete autoantibodies against glomerular antigens have been isolated from nephritic MRL/*lpr* mice, and MRL/*lpr* and NZB/W F1 mice with established nephritis also exhibited increased intra-renal B cells and plasma cells infiltration [67,68]. In NZB/W F1 mice, intra-renal plasma cells showed comparable number and immunoglobulin secretion as the plasma cells residing in the bone marrow, and the degree of intra-renal plasma cell infiltration also correlated with anti-dsDNA autoantibodies titers [68]. The pathogenic contribution of the B cell repertoire in LN extends beyond autoantibodies production. While glomerulonephritis can still develop in MRL/*lpr* mice which were genetically manipulated to become incapable of antibody secretion, severe nephritic lesions do not occur in MRL/*lpr* mice with B-cell deficiency and such observation might be related to impaired development of activated cytotoxic and helper T cells in these mice [69,70]. In SLE patients, there was an increase in the frequency of peripheral plasma cells and memory B cells but diminished number of circulating naïve B cells [71,72]. Such alterations in the B cell subsets profiles have several important implications on disease behavior in SLE and LN patients. Memory B cells have reduced FcγRIIb expression and thus lower reactivation threshold [72,73]. The low proliferation rates in memory B cells also make them less susceptible to conventional immunosuppressive medications which are cell-cycle dependent, and thus become more readily reactivated during disease relapse [71]. Patients with active SLE have elevated number of circulating plasma cells, which shows positive relationship with serum levels of immunoglobulin and anti-dsDNA autoantibodies and disease activity scores [71,74]. Furthermore, the degree of intra-renal plasma cell infiltration also correlates with renal histological activity and chronicity indices in LN patients [68]. 

## 5. Effect of Immunosuppressive Treatments on B Cells and Implications on the Choice of Therapies in Lupus nephritis

**Figure 1:**
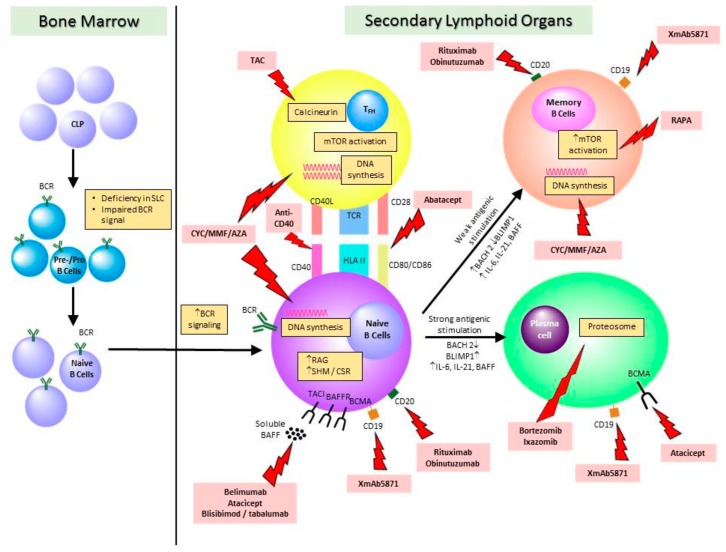
Schematic diagram showing the B cell abnormalities in systemic lupus erythematosus and the potential therapeutic targets.

Treatment for LN is generally governed by histopathological findings and the severity of renal disease. Immunosuppressive treatment is indicated in patients presenting with active severe LN—focal or diffuse proliferative (Class III or IV) LN, or severe membranous (Class V) LN. Clinical management is often divided into the induction and maintenance phases. The induction phase entails the use of high-dose immunosuppressive therapies for approximately four to six months, with the goal to abate active renal inflammation and limit kidney parenchymal damage. The maintenance phase refers to the application of continuous low-dose immunosuppression after induction therapy, to consolidate response and to prevent disease relapse. Given its instrumental roles in SLE and LN pathogenesis, the B cell repertoire is therefore an attractive therapeutic target in SLE and LN (Figure 1) and the effect of immunosuppressive treatments on B cells have important clinical implications (Table 1).

### 5.1. Conventional Immunosuppressive Treatments for SLE and LN

The current standard-of-care induction treatments for active severe LN are high-dose corticosteroids combined with either cyclophosphamide (CYC) or mycophenolate (MMF), and the maintenance therapies are low-dose corticosteroids coupled with either MMF or azathioprine (AZA) [75,76,77,78]. The current data show that CYC or MMF induction conferred similar short-term efficacy in patients with active proliferative LN [79,80,81,82]. Interestingly, MMF induction was associated with earlier reduction of circulating plasmablasts and plasma cells compared with CYC [83]. Also, CYC induction showed preferential depletion of less mature B cells such as naïve B cells and pre-switched memory B cells compared with MMF [83]. Whether these effects on B cell subsets can account for the better comparative efficacy of MMF in some high-risk ethnic groups (e.g., Afro-Americans) remain speculative. Other investigators have reported that CYC or MMF induction in active SLE patients was associated with different T cell subset profiles but not the B cell subpopulations after 4 weeks of treatment [84]. More importantly, neither CYC nor MMF had significant effects on class-switched memory B cells which are typically resting and non-proliferating during active LN and therefore these memory B cells can be activated to trigger disease relapse in patients who have apparently responded well to induction treatments [83]. Accumulating evidence has demonstrated MMF maintenance is associated with lower risk of disease relapse compared with azathioprine (AZA) maintenance [85,86,87]. Indeed, data from in vitro studies suggested that the AZA dose required for the suppression of humoral response in mice was significantly higher than that required for the inhibition of cellular immunity [88,89]. It has also been demonstrated that SLE patients receiving MMF maintenance showed lower number of circulating plasmablasts but higher number of naïve and transitional B cells compared with patients receiving AZA maintenance [90]. The authors also showed that MMF could profoundly suppress the proliferation of B cells from healthy individuals in vitro, but there was no comparison with AZA, and no information on lupus B cells [90]. Our preliminary results showed decreased miRNA-148a and increased BACH1, BACH2, and PAX5 expression in B cells from LN patients receiving MMF maintenance treatment compared with B cells from AZA-treated patients [91,92]. The B cells from MMF-treated LN patients also showed reduced cell proliferation upon ex vivo stimulation compared with B cells from patients receiving AZA maintenance [92]. These differences in B cell subset profiles, cellular signatures, and proliferative capacity provide a rationale for the relatively lower relapse rate in patients receiving MMF maintenance observed clinically.

### 5.2. Biologics and Emerging Therapies or SLE and LN

Anti-CD20 monoclonal antibody is an established treatment for B cell malignancy and has also been used in various autoimmune conditions [93,94]. CD20 is a cell surface marker expressed on different B cell subpopulations, and therefore anti-CD20 treatment can lead to profound depletion of B lymphocytes which usually last for at least 6 months [95]. The mechanisms of B cell depletion with anti-CD20 monoclonal antibodies include antibody-dependent cell-mediated cytotoxicity (ADCC), complement-dependent cytotoxicity, and enhanced apoptosis of B cells [96]. Treatment of MRL/*lpr* mice with anti-CD20 monoclonal antibodies resulted in decline of anti-dsDNA autoantibodies and amelioration of renal lesions [97]. Also, administration of anti-CD20 monoclonal antibodies in NZB/W F1 mice could delay the onset of disease and retard progression of glomerulonephritis [98]. Rituximab is a chimeric monoclonal antibody against CD20, which was initially developed for the treatment of B cell lymphomas [93]. The effect of rituximab treatment on B cells is quite variable between SLE patients [99,100,101]. Previous studies showed that rituximab treatment could lead to profound B cell depletion as early as 2 weeks after administration, and B cells repopulate to approximately 65% of the baseline levels after 12 months [102,103]. Results from the LUNAR trial showed that rituximab as add-on therapy to standard induction treatments did not improve renal outcomes although the rituximab group showed more improvement in the levels of complement and anti-dsDNA and proteinuria [104]. The failure to achieve additional renal benefit may be related to the potent background immunosuppressive treatments, and that anti-CD20 treatment only deplete circulating B lymphocytes but not tissue B cells and plasma cells which do not express CD20. While the clinical response to anti-CD20 treatment could be quite variable between SLE patients, monitoring of B cell biomarkers might help predict treatment response and disease course. Persistent presence of B cells was associated with poor therapeutic response, and early repopulation of memory B cells and plasmablasts was related to early disease relapse [101]. Lower pre-treatment plasmablasts were predictive of complete B cell depletion, and B cell depletion at 6 weeks increased the odds of major clinical response [105]. Other investigators also reported that faster repopulation of B cells was associated with early relapse, and patients who relapsed with high anti-dsDNA levels showed increased percentage of circulating IgD^-^CD27^hi^ plasmablasts whereas those with low anti-dsDNA levels was accompanied by a higher percentage of circulating IgD^-^CD27^-^ B cells [106]. Nevertheless, anti-CD20 monoclonal antibodies have roles in refractory or frequently relapsing SLE, severe extra-renal lupus complications and steroid minimization [107,108,109,110]. The ACR guidelines also recommends that rituximab treatment in LN patients who fails to improve or worsens after 6 months of standard induction therapies, or after the patient has failed both CYC or MMF treatments [75]. The latest EULAR guideline 2019 suggests that rituximab is to be considered in SLE patients who have severe renal or extra-renal (mainly hematological and neuropsychiatric) disease refractory to other immunosuppressants and/or belimumab, or in patients with contraindications to other immunosuppressive drugs [111]. One recent phase 2 study (NOBILITY, NCT02550652) demonstrated that obinutuzumab, a new Type 2 monoclonal antibody with enhanced ADCC, in combination with corticosteroids and MMF met its primary and secondary efficacy endpoints for active proliferative LN, and a phase 3 clinical trial will be conducted shortly to verify these encouraging observations [112]. Depletion of B lymphocytes can also be achieved by targeting other B cell surface molecules such as CD19 (XmAb5871) [113] and CD22 (epratuzumab) [18,114,115], and further clinical data are required to establish their roles in SLE and LN patients. Proteosome inhibitors (PI) can effectively deplete plasma cells and hence are potentially useful treatments in SLE and LN patients. Preliminary clinical data showed that bortezomib could significantly deplete peripheral and bone marrow plasma cells and improved proteinuria and serological parameters in active LN patients who were refractory to standard induction treatments [116,117]. However, there was a high rate of treatment discontinuation due to adverse reactions. Bortezomib is currently being tested in a Phase 2 trial for SLE (NCT02102594) and ixazomib (a second-generation oral PI with less neurological side effects) is being investigated in LN patients (NCT02176486). The application of chimeric antigen receptor T cells (CAR-T) technology can potentially confer more profound and sustained B cell depletion, and is shown to be highly effective in a murine lupus model [118].

Inhibition of B cell survival factors is another therapeutic approach in SLE and LN. Treatment of NZB/W F1 mice with a soluble TACI-Ig or BAFF receptor-Ig fusion protein could suppress proteinuria and improve survival [22,119]. Belimumab is a humanized IgG1γ monoclonal antibody against soluble BAFF. One prospective cohort study showed that belimumab treatment was associated with early reduction of both naïve and autoimmunity-associated B cells (CD11c + CD21-) at 3 months, but with little impact on class-switched memory B cells and plasma cells over a follow-up duration of 3 years [120]. Results from two randomized controlled trials (RCT) (BLISS-52 and BLISS-76) showed that belimumab treatment was effective in active SLE patients, but patients with moderate to severe nephritis were excluded from these two studies [121,122]. Post hoc analysis of the pooled data from these two RCTs suggested that patients treated with belimumab showed reduced risk of renal flare [123], and therefore another RCT (BLISS-LN; NCT01639339) is underway to investigate the use of belimumab in active LN patients. Substantial and continual reduction of B lymphocyte subsets was observed beyond 76 weeks of belimumab treatment, with 80–90% reduction for naïve plasmacytoid B cells, 70–75% decrease for CD19+/CD20+ B cells, and 50–60% drop for plasma cells respectively [124]. Memory B cells showed a biphasic response to belimumab, with a rapid increase through week 8, possibly as a result of transient redistribution of these cells from lymphoid tissues to the circulation, then followed by a gradual decline [124]. Whether the lower risk of renal flares in patients receiving belimumab was associated with these changes in B cell repertoire remains speculative. Although data on belimumab in SLE and LN appeared to be quite encouraging, clinical trials on other anti-BAFF therapies showed conflicting results. Tabalumab is a monoclonal antibody targeting both soluble and membrane-bound BAFF. The results from two Phase 3 RCTs (ILLUMINATE-1 and -2) demonstrated that tabalumab treatment did not reach the primary endpoints in active non-renal SLE patients [125,126]. Post hoc analysis of the pooled data from ILLUMINATE-1 and -2 also did not reveal any benefits on renal outcomes [127]. Furthermore, the inhibition of B cell growth factors in addition to standard induction therapies should also be used with caution, as one previous Phase 2/3 study on atacicept (a human recombinant fusion protein of TACI and the Fc portion of IgG1) had early discontinuation due to significant risk of hypogammaglobulinemia and infections [128]. Other new biologics targeting the BAFF/APRIL axis include blisibimod (NCT01395745), RC18 (TACI-antibody-fusion protein; NCT02885610), AMG570 (bispecific peptibody against BAFF), and ICOS ligand (NCT02618967). In this regard, data from a phase 3 randomized, double-blind, placebo-controlled trial suggested that blisibimod did not meet its SRI-6 endpoint but there was significantly higher proportion of patients in the treatment arm achieved partial renal response and corticosteroids taper [129].

Interruption of B cell–T cell interaction represents another novel therapeutic strategy in SLE and LN. Abatacept is a fusion protein of CTLA-4 linked to the Fc portion of IgG1 and can selectively intervene the CD28-CD80/86 costimulatory signal, and thereby attenuate B cell–T cell interactions. In vitro studies showed that abatacept treatment would decrease CD80/CD86 expression and impair memory formation in human B lymphocytes [130]. Inhibition of the co-stimulatory molecule has been shown to be effective in murine models of LN [131,132,133]. Abatacept is currently approved for the treatment of rheumatoid arthritis and juvenile inflammatory arthritis [134]. Previous studies in RA patients demonstrated that treatment with abatacept was associated with selective reduction of memory B cells, and higher basal memory B cells counts were predictive of good treatment response [135]. However, active Class III or IV LN patients treated with abatacept on a background of corticosteroids and MMF showed improvements in serological markers and proteinuria but did not achieve the primary study endpoint of improving renal outcomes [136]. The negative result might be related to the potent background therapy and stringent definitions of complete renal response [137]. Nevertheless, abatacept treatment may potentially confer long-term benefits in LN patients because the selective depletion of memory B cells may decrease the risk of subsequent renal relapses. Blockade of CD40/CD40L pathway also represents another innovative approach to modulate B cell–T cell interaction and a humanized anti-CD40 monoclonal antibody (CFZ533) is now being studied in LN patients (NCT03610516). While anti-IL-6 treatment was effective in murine LN [47,48], in a phase 2 RCT anti-IL-6 treatment (PF-04236921) did not achieve the primary efficacy endpoint in patients with non-renal SLE and the higher dose (200 mg) cohort was discontinued due to safety issues [138].

Calcineurin inhibitors (CNI) and mammalian target of rapamycin (mTOR) inhibitors are emerging treatments for SLE and LN. Calcineurin inhibitors show specific suppressive actions on T lymphocytes and hence do not affect B lymphocytes directly. Instead, the effects of CNI on B cells are mediated through T cell-dependent manners such as inhibition of T_FH_ and germinal center reactions [139,140,141]. Tacrolimus (TAC) is a CNI which has most clinical data on the management of LN to date, and current experience suggests that TAC when used in dual or triple immunosuppression showed at least comparable efficacy as CYC- or MMF-based induction and with earlier reduction of proteinuria [142,143,144,145]. A recent phase 2 study reported that a new CNI voclosporin, when combined with corticosteroids and MMF, resulted in higher renal response rates in active LN patients compared with placebo [146]. There is little clinical data on the effect of CNI on B cell biology in SLE and LN patients. Sirolimus (RAPA) is an mTOR inhibitor with suppressive effects on both B and T lymphocytes. One in vitro study showed that RAPA treatment could effectively inhibit human memory B cells proliferation and plasma cell differentiation [140]. Another recent study reported that pathogenic memory B cells isolated from treatment-naïve SLE patients showed increased mTORC1 activation, and the proliferation of these memory B cells can be significantly suppressed by RAPA treatment in vitro [147]. Furthermore, treatment of NZB/W F1 mice with RAPA could prevent development of nephritis and alleviate established nephritis, which was also accompanied by reduction in intra-renal B cell infiltration [148,149]. Whilst clinical data on the use of RAPA in patients with SLE and LN continue to emerge [150,151,152], its effect on B lymphocytes remains to be fully understood.

## 6. Future Directions and Concluding Remarks

The B cell repertoire shows pathogenic significance in SLE and LN, and therefore targeting B cells and plasma cells presents an appealing therapeutic approach in SLE and LN. Increasing understanding of B cell biology in SLE and LN has propelled the development of novel and more specific treatments. While many drug development programs for B cell-targeted therapies have been apparently unsuccessful either due to failure to achieve primary endpoints or because of excessive adverse events, future studies are still warranted. In this regard, attention to study design such as modification in background therapies or study endpoints, or more selective recruitment of subjects based on aberrant B cell signatures may confer a higher chance of success and inform the selection of patients for these novel therapies.

Within the bone marrow, deficiency in surrogate light chain (SLC) and impaired BCR signaling at the stage of pre-/pro-B cells will enhance survival of autoreactive B cells which contribute to the pathogenesis of systemic lupus erythematosus and lupus nephritis. In the secondary lymphoid organs (e.g., spleen or lymph nodes), naïve B cells will interact with follicular T helper (T_FH_) cells and further differentiate into memory B cells or plasma cells. At this stage, mechanisms which augmented the pathogenicity of B cell repertoire in SLE and LN include enhanced RAG expression and BCR signaling, increased somatic hypermutation (SHM) and class-switch recombination (CSR), aberrations in B cell transcription factors (e.g., BACH2, BLIMP1) and increased expression of B cell-related cytokines (e.g., BAFF, IL-6 and IL-21). Ways to affect the B cell repertoire include disruption of DNA synthesis (e.g., cyclophosphamide, mycophenolate mofetil, azathioprine), direct cell depletion by binding to cell surface molecules (e.g., rituximab, obinutuzumab), neutralization of B cell survival factors (e.g., belimumab, atacicept), interruption of B cell–T cell interaction (e.g., abatacept), inhibition of T cell (tacrolimus) activation and mTOR signaling (sirolimus), and inhibition of proteasome in plasma cells (e.g., bortezomib, ixazomib).

AZA, azathioprine; BAFF, B cell activating factor; BAFF-R, BAFF receptor; BCR, B cell receptor; BCMA, B-cell maturation antigen; CLP, common progenitor precursors; CSR, class-switch recombination; CYC, cyclophosphamide; mTOR, mammalian target of rapamycin; MMF, mycophenolate mofetil; RAPA, sirolimus; SHR, somatic hypermutation; TAC, tacrolimus; TACI, transmembrane activator and calcium modulator and cyclophilin ligand interactor TCR, T cell receptor; T_FH_, follicular T helper cells.

## Figures and Tables

**Table 1:** The effect of currently available immunosuppressive medications on B cells.

Drugs	Mechanisms of Action	Effect on B Cells
CYC	Disrupts DNA replication and thus confers cytotoxic effect on actively proliferating cells including lymphocytes	Preferentially depletes less mature B cells (e.g., naïve B and pre-switched memory B cells) compared with MMF. Little effect on class-switched memory B cells
MMF	Inhibits IMPDH and therefore selectively blocks de novo purine synthesis in B and T lymphocytes	Earlier reduction of circulating plasmablasts compared with CYC but with little effect on class-switched memory B cells More potent than AZA in suppressing naïve and memory B cell proliferation
AZA	Converts to 6-mercaptopurine and interferes with DNA replication and purine synthesis in lymphocytes	Animal data shows higher AZA dose required to suppress humoral immunity than that required to suppress cellular immunity
TAC	Inhibits IL-2 production and thus T cell activation and proliferation	Inhibits T_FH_ and GC formation, thereby impairs B cell maturation and antibody production
RAPA	Inhibits the activation of mTOR signals in lymphocytes	Suppresses proliferation of different B cell subsets (especially memory B cell with ↑mTORC1 activation). Blocks differentiation of B cells into plasma cells. ↓intra-renal B cell infiltration in murine LN models
Rituximab	Binds to CD20 on B cells, leading to ADCC, CDC and ↑apoptosis of B cells	Profoundly depletes different B subsets except plasma cells within 2 weeks. B cell reconstitution occurs at approximately 6–9 months
Belimumab	Inhibits BAFF and hence survival and maturation of B cells	Sustained reduction in naïve plasmacytoid B cells (80–90%), CD19+/CD20+ B cells (70–75%) and plasma cells (50–60%).
Abatacept	Interruption of co-stimulatory signal for B cell–T cell interaction	Preferentially suppresses memory B cells

ADCC, antibody-dependent cell-mediated cytotoxicity; AZA, azathioprine; BAFF, B cell activating factor; CDC, complement-dependent cytotoxicity; CYC, cyclophosphamide; GC, germinal center; IL-2, interleukin-2; IMPDH, inosine monophosphate dehydrogenase; MMF, mycophenolate mofetil, mTORC, mammalian target of rapamycin; RAPA, sirolimus; TAC, tacrolimus; TFH, follicular T helper cells. ↑: increase; ↓: decrease.
